# Depression in mothers of children with thalassemia or blood malignancies: a study from Iran

**DOI:** 10.1186/1745-0179-2-27

**Published:** 2006-10-04

**Authors:** Afshan Sharghi, Mojgan Karbakhsh, Behrooz Nabaei, Alipasha Meysamie, AliReza Farrokhi

**Affiliations:** 1 Department of Community Medicine, Ardebil University of Medical Sciences, Daneshgah Ave, Ardebil, Iran; 2 Department of Community Medicine, Tehran University of Medical Sciences, Poursina st, Qods st, Enqelab st, Tehran, Iran; 3 Department of Community Medicine, Tehran University of Medical Sciences, Poursina st, Qods st, Enqelab st, Tehran, Iran; 4 Legal Medicine Center of Ardebil Province, Panzdah Khordad Square, Ardebil, Iran

## Abstract

**Background:**

Several studies have found that parents of children with chronic diseases or disabilities have higher depression scores than control parents. Mothers usually take on the considerable part of the extra care and support that these children need and thus are at markedly increased risks of suffering from psychological distress and depression. The main aim of the present study was to investigate if mothers of children with thalassemia or blood malignancies have higher scores of depression compared with a group of control mothers.

**Methods and materials:**

In this cross – sectional study, 294 mothers were recruited in three groups and assessed using the Beck Depression Inventory (BDI): mothers of children with thalassemia, mothers of children with blood malignancies and a control group. SPSS version 11.5 with chi square, ANOVA, linear and logistic regression were used for statistical analysis.

**Results:**

The only variable bearing a statistically significant relationship with the depression score of mothers was the child's disease: for thalassemia with OR of 2.17 (95% CI = 1.16–4.0, P = 0.015), for blood malignancies with OR of 2.71 (95% CI = 1.48–4.99, P = 0.001).

**Discussion and conclusion:**

The results of this study can contribute to the development of a screening program for decreasing depression burden and promoting quality of life for mothers of children with thalassemia or blood malignancies.

## Background

In every day use of language, the term "depression" is meant to describe different feelings such as sadness, disappointment, hopelessness and abatement. In fact, depression is a psychiatric disorder in which patient's mood becomes distressed. Today, undiagnosed and untreated depressions have become major health problems throughout the world. Nowadays, depression is so common that it is sometimes called "psychological Flu" [1]. In several studies, the prevalence of depression has been reported to be about 10 to 30 percent [1,2].

On the basis of World Health Organization reports on burden of diseases, depression is on the top regarding the extent of its related disability and death: the suicide risk is 30 times higher in depressed people compared with the normal population [3].

In many studies, prevalence of depression in women is reported to be two times more than men and it is attributed to their biologic, psychological and socioeconomic conditions [1,4,5]. Women have to deal with multiple roles inside and outside of the house which are sometimes opposing [6]. On the other hand, mothers' depression may decrease their sense of responsibility regarding taking care of their children and getting involved in their education and proper nutrition. It may also give rise to rejecting behavior and hostility toward children [7-9]. In brief, depression affects almost all aspects of life and can eventually make normal life impossible. This is while many types of depression are amenable to treatment and the more the treatment is delayed the harder it becomes to treat the patient [1].

It seems that depressive people experience specific events before depression. These include monetary concerns, difficulty with spouse, parents or children and sometimes physical problems. Several studies have indicated that presence of a patient or disabled child in the family can cause stress and depression in parents[10-14].

Mothers usually take active roles in the care of their ill children and even might quit their jobs or favorite activities. Thus, they bear greater stresses than fathers and are at higher risks of depression [15].

In the year 2000, Kaviani and colleagues employed BDI test and clinical psychiatric interview on 1070 adults in a random sample of Iranian population living in the capital city of Tehran. They showed that overall prevalence of depressive disorders in Tehran was about 22.5 percent: 30.5 percent among women (mean BDI score = 12.16) and 16 percent among men(mean BDI score = 8.47). In that study, there appeared to be a significant relationship between depression and educational level, marital status, and occupation [2].

In another study from Iran performed on residents of medical specialty courses at Tehran University of Medical Sciences, the relationship between life events and depression was investigated. According to Beck Depression Inventory criteria and the Holmes & Rahe Life Events Scale, 19.3 percent of study subjects were depressed. The average number of life events before depression was 6.5 which was significantly related with depression. In that study, no significant correlation was detected between depression and age, sex, marital status, number of children and religion. Men were more sensitive to occupational and financial problems as predisposing factors to depression; while, personal and marital problems were more commonly implicated for women[16].

Olsson et al, compared the extent of depression in parents of children suffering from autism or intellectual disabilities with control group. According to Beck Depression Inventory, these parents had higher scores for depression. He quotes from other studies that the prevalence of depression among parents of disabled children to be between 35 to 53 percent [15].

Blood disorders are not uncommon in the children. Blood malignancies- for instance- are the second most common malignancies in children. In Iran, there are about 20,000 thalassaemic patients and 3,000,000 carriers. In our country, the average birth rate of infants with major beta thalassaemia is 0.73/1000 live births, ranging from 0.08 – 3.52/1000 by province [17].

Thalassemic patients need monthly blood transfusions and regular iron chelation for survival; while children with blood malignancies are treated with chemotherapy protocols and sometimes bone marrow transplantation. Thus, parents of these children are exposed to protracted physical and emotional suffering for their offsprings' devastating health problem. They usually feel the most responsible, guilty and hopeless, as well as worried about the health and future of their children[10,11].

This study is among the first investigations addressing the issue of relationship between life long or life threatening hematologic diseases in children and depression among their mothers. We aimed to answer the following questions:

1) Do these mothers obtain higher scores for depression than mothers in control group? and 2) Is there any relationship between mothers' depression score and variables such as age, education, marital status, problems with husband, problems with own family or husband's family, previous history of depression in family and death of parents before the age of twelve?

## Materials and methods

In this cross sectional study, the participants were mothers referring to Children Hospital Medical Center(the major pediatric hospital of Tehran University of Medical Sciences) in winter 2004, to receive treatment for their children(age < 15). Mothers who consented to participate in this project were included and were recruited in three groups; first, mothers of children with thalassemia, second; mothers of children with blood malignancies and third, control group consisting of mothers of children without any blood malignancies or thalassemia referring to general clinic of the hospital for more general problems.

A sample size of 95 was calculated(for each group) with the following formula: $n=\frac{2{\left(z1-\frac{\alpha }{2}+Z\beta \right)}^{2}P(1-P)}{{\left(P1-P2\right)}^{2}}$ considering type one error(α) of 0.05 and power of 0.8 and p1 and p2 equal to 0.3 and 0.5, respectively.

Questionnaires used for data collection consisted of two parts: the first part was completed by interviewers and had 22 questions (19 about the mother and 3 about the child). The second part – a translated and validated version of Beck 's depression Inventory- consisted of 21 questions and was filled in by the mothers, themselves.

Beck Depression Inventory (BDI) is one of the self -administered tests which is used as an objective method for measuring the intensity of depressive symptoms. In this study mothers with scores equal to or higher than 19 were considered depressed [18]. SPSS version 11.5 with chi square, ANOVA, linear and logistic regression was used for statistical analysis.

## Results

In this study, 294 mothers including 98 from first group (mothers of thalassemic children), 97 from the second group (mothers of children with blood malignancies) and 99 from third group (controls) were studied.

Demographic characteristics of mothers in these three groups are summarized in table 1.

**Table 1:** Demographic characteristics of mothers in the three groups

Characteristic	Group1*	Group2**	control***	total	P value
Age of the subjects(μ ± δ)	36.2 ± 7.8	32.7 ± 6.1	29.9 ± 6.5	32.1 ± 7.3	0.014¶
Age of their children(μ ± δ)	7.1 ± 5.9	7.5 ± 4.5	7.7 ± 4.8	7.4 ± 5.3	> 0.05
Marital status of the mother(%)					
Married	94.9	94.9	96	95.3	> 0.05
Divorced	3	1	1	1.7	
widow	2	4	3	3	
					
Educational Status					
					
illiterate	10.6	7.2	8.1	8.6	> 0.05
Primary education	26.6	41.2	24.2	30.7	
Guidance school	25.5	24.7	28.3	26.2	
Diploma	30.9	24.7	33.3	29.7	
Associate diploma^§^	1.1	2.1	1	1.4	
BS/BA	5.3	0	5.1	3.4	
					
Living status of mothers' parents at the age of 12(%)					
					
Both parents alive	90	85	85.9	87	> 0.05
					
History of depression (%positive)	13	19.2	14.1	15.4	> 0.05
					
Family History of depression (%positive)	12	12.2	14.3	12.8	> 0.05
					
Conflict with husband(%positive)	14.1	8.2	15.5	12.6	> 0.05
					
Conflict with own or husband's family(%positive)	20	16.2	20.2	18.8	> 0.05

* Mothers of thalassemic children,

** Mothers of children with blood malignancies,

*** Controls

¶ Statistically significant difference

§ (2 years of college study)

The mean BDI score for all mothers was 17.23 (SD = 10.8) which was 17.9 (SD = 10.6) for the first group, 20(SD = 11.3) for the second group and 13.81 (SD = 9.6) for the control group. There was also significant difference between first and second groups with control group(P = 0.01); while, there wasn't any statistical difference between the first and second groups.

Also, in classifying the groups according to BDI score,46.3 percent of all of the mothers, 56.7 percent of mothers of children with malignancy (group 2), 51 percent of mothers of thalassemic children and 31.4 percent of control group had BDI score higher than 18 and there was a significant statistical difference between first and second group with third group (P = 0.003).

Regarding the relationship between mean BDI scores and study variables, an inverse statistical association was found between mother's age and BDI score (P = 0.001). Table 2 demonstrates the mean BDI scores according to other variables under study.

**Table 2:** The relationship between mean BDI scores and study variables among mothers studied

Variable	Beck Depression Inventory score (Mean ± sd)	P value
Marital status		
		
Married	16.9 ± 10.8	
Divorced	28.6 ± 9.6	> 0.05
widow	22.0 ± 7.1	
		
Educational Status		
		
Illiterate	19.8 ± 10.2	
Primary education	19.1 ± 11.7	
Guidance school	15.9 ± 10.6	> 0.05
Diploma	15.2 ± 8.7	
Associate diploma	22.5 ± 15.1	
BS/BA	17 ± 17.2	
		
Living status of (mothers') parents at the age of 12		> 0.05
		
Mother passed away, father alive	21.8 ± 8.1	
Father passed away, mother alive	18.3 ± 11.6	
Both parents passed away	36.5 ± 17.6	
Both parents alive	16.9 ± 10.6	
		
Subjects with positive past history of depression	22.5 ± 2.1	> 0.05
Subjects with positive family history of depression	20.4 ± 14.2	> 0.05

In logistic multivariate regression analysis, the only variable bearing a statistically significant relationship with the depression score of mothers was the child's disease: for thalassemia with OR of 2.17 (95% CI = 1.16–4.0, P = 0.015), for blood malignancies with OR of 2.71 (95% CI = 1.48–4.99, P = 0.001).

## Discussion

Existence of life threatening or long-lasting diseases in children is a condition which causes stress for mothers and can predispose them to depressive disorders. In our study, the depression score in mothers of thalassemic children and children with malignancies was significantly higher than mothers in the control group. In the study of Olsson from Sweden, 45 percent of mothers having children with intellectual disorders, 50 percent with children suffering from autism and 17 percent of the control group were depressed according to Beck depression Inventory [15].

In our series, the prevalence of depression was higher among mothers children afflicted with blood disorders than mothers in control group, which was similar to other reports [15]. Importantly enough, the prevalence of depression among our controls was higher than that in the control group of Olsson's study. This might be partially explained by the following reasons: as hospital-based controls, we selected mothers who had come themselves or had been referred to a university hospital seeking treatment for their children. Thus this study has the shortcomings of using hospital versus community controls (they might have been of low socioeconomic class; the children's disease might have been refractory to general managements, etc). Unfortunately, we can not quantify this as we don't have access to other characteristics of this control group eg the reason for admission, etc.

According a previous study from Iran, the mean BDI score for women in Tehran has been 12.16[2] which is slightly lower than BDI score of our control group; but, is remarkably lower than scores of the other two groups of our series. This can also indicate the role of child's disease on the depression score of the mother.

In another research report from Torronto, the mean BDI scores of women referring to a psychotherapy center has been 23.6 (SD = 12) which is close to the BDI score of mothers of children suffering from blood malignancies in our study. This similarity in scores can implicate the unmet need of these mothers for receiving psychiatric services [19].

We found out that thalassemia and blood malignancies were related to mother's depression with the odds ratio of 2.17 and 2.71, respectively. In a cohort study from Sweden, a significant relationship between malignancies in children and mother's depression was also detected (RR = 1.4), which also insist that child's disease can affect mother's depression level [20].

In our study, the only variable showing independent relationship with mother's depression score was life-long or life threatening blood disorders in the child. Mother's age was related in univariate analysis; but not in multivariate logistic regression, which is in accordance with previous studies [2,16].

The relationship between mothers' education and BDI score showed a trend similar to some previous studies: with educational levels up to high school certificate, higher education was related with lower scores; while, among university educated mothers, the relationship was just the opposite of what previously said. Nevertheless, the trend did not prove to be statistically significant. It can been hypothesized that educated mothers can cope better with the children's illness; but, more responsibilities or insight of college-educated mothers regarding the child's devastating condition may predispose them to depression.

Divorced mothers had higher score than the other two groups(widows and married mothers). This result was also similar with previous studies.(5,21). Marriage and living with husband is an example of social support and can be related with lower depression scores.

Long-lasting or life-threatening diseases of children, especially those which don't have definitive cures are important factors inducing stresses to mothers and can put them at conflicts with life situations and increase the risk of depression.

In medical centers for the care of chronically/seriously ill children, there should be some units providing psychiatric consultation in order to screen and manage depressive disorders among mothers. This can help them get through the child's disease more healthily and hopefully and let them remain effective caregivers for the ill child and also for the rest of the family.
